# Supplementary material for: Detecting sequence signals in targeting peptides using deep learning
Source: Life Sci Alliance. 2019 Sep 30;2(5):e201900429. doi: 10.26508/lsa.201900429 (PMC6769257; doi:10.26508/lsa.201900429)
Supplement: Supplementary file 1 [file LSA-2019-00429_TableS1.docx]

Table S1: F1 score for the MLP predictor, using different number of N-terminal residues.

| \|  \| Tool \| SP \| mTP \| cTP \| luTP \| noTP \| Average \| \| --- \| --- \| --- \| --- \| --- \| --- \| --- \| --- \| \|  \| MLP-5 \| 0.57 \| 0.22 \| 0.03 \| 0.00 \| 0.88 \| 0.77 \| \|  \| MLP-10 \| 0.80 \| 0.55 \| 0.33 \| 0.04 \| 0.93 \| 0.87 \| \|  \| MLP-15 \| 0.90 \| 0.57 \| 0.42 \| 0.00 \| 0.95 \| 0.91 \| \|  \| MLP-20 \| 0.93 \| 0.63 \| 0.43 \| 0.04 \| 0.96 \| 0.93 \| |
| --- | --- | --- | --- | --- | --- | --- | --- | --- | --- | --- | --- | --- | --- | --- | --- | --- | --- | --- | --- | --- | --- | --- | --- | --- | --- | --- | --- | --- | --- | --- | --- | --- | --- | --- | --- | --- | --- | --- | --- | --- |
