# Supplementary material for: Detecting sequence signals in targeting peptides using deep learning
Source: Life Sci Alliance. 2019 Sep 30;2(5):e201900429. doi: 10.26508/lsa.201900429 (PMC6769257; doi:10.26508/lsa.201900429)
Supplement: Supplementary file 2 [file LSA-2019-00429_TableS2.docx]

Table S2: Confusion matrix for non-plant organisms representing the number of proteins for each targeting peptides predicted by TargetP 2.0 (rows) versus observed in the test set (columns).

| \|  \| Class \| SP \| mTP \| noTP \| \| --- \| --- \| --- \| --- \| --- \| \|  \| SP \| 2390 \| 5 \| 47 \| \|  \| mTP \| 2 \| 311 \| 44 \| \|  \| noTP \| 23 \| 58 \| 7644 \| |
| --- | --- | --- | --- | --- | --- | --- | --- | --- | --- | --- | --- | --- | --- | --- | --- | --- | --- | --- | --- | --- |
