# Supplementary material for: Detecting sequence signals in targeting peptides using deep learning
Source: Life Sci Alliance. 2019 Sep 30;2(5):e201900429. doi: 10.26508/lsa.201900429 (PMC6769257; doi:10.26508/lsa.201900429)
Supplement: Supplementary file 3 [file LSA-2019-00429_TableS3.docx]

Table S3: Confusion matrix for Viridiplantae representing the number of proteins for each targeting peptides predicted by TargetP 2.0 (rows) versus observed in the test set (columns).

| \|  \| Class \| SP \| mTP \| cTP \| luTP \| noTP \| \| --- \| --- \| --- \| --- \| --- \| --- \| --- \| \|  \| SP \| 272 \| 0 \| 2 \| 0 \| 3 \| \|  \| mTP \| 0 \| 117 \| 2 \| 0 \| 11 \| \|  \| cTP \| 0 \| 2 \| 197 \| 9 \| 9 \| \|  \| luTP \| 0 \| 0 \| 11 \| 34 \| 0 \| \|  \| noTP \| 10 \| 6 \| 15 \| 2 \| 1779 \| |
| --- | --- | --- | --- | --- | --- | --- | --- | --- | --- | --- | --- | --- | --- | --- | --- | --- | --- | --- | --- | --- | --- | --- | --- | --- | --- | --- | --- | --- | --- | --- | --- | --- | --- | --- | --- | --- | --- | --- | --- | --- | --- | --- |
