# Supplementary material for: Detecting sequence signals in targeting peptides using deep learning
Source: Life Sci Alliance. 2019 Sep 30;2(5):e201900429. doi: 10.26508/lsa.201900429 (PMC6769257; doi:10.26508/lsa.201900429)
Supplement: Supplementary file 4 [file LSA-2019-00429_TableS4.docx]

Table S4: Performance of TargetP 2.0 considering only the peptide prediction in one kingdom at a time. This table shows the performance in the test set yield by each predictor for Mitochondria (mTP), Chloroplast (cTP) , Thylakoid (luTP), Signal Peptide (SP), and the other proteins without targeting peptide (noTP), in terms of F1 score, Matthews correlation coefficient (MCC), Precision and Recall

| \|  \| Kingdom \| Loc \| Proteins \| Precision \| Recall \| F1-Score \| MCC \| \| --- \| --- \| --- \| --- \| --- \| --- \| --- \| --- \| \|  \| Viridiplantae \| SP \| 282 \| 0.98 \| 0.96 \| 0.97 \| 0.97 \| \|  \| Metazoa \| SP \| 2251 \| 0.98 \| 0.99 \| 0.99 \| 0.98 \| \|  \| Fungi \| SP \| 133 \| 0.91 \| 0.99 \| 0.95 \| 0.95 \| \|  \| Other \| SP \| 31 \| 1.0 \| 0.97 \| 0.98 \| 0.98 \| \|  \| Viridiplantae \| mTP \| 125 \| 0.9 \| 0.94 \| 0.92 \| 0.91 \| \|  \| Metazoa \| mTP \| 263 \| 0.89 \| 0.86 \| 0.87 \| 0.87 \| \|  \| Fungi \| mTP \| 103 \| 0.83 \| 0.77 \| 0.80 \| 0.79 \| \|  \| Other \| mTP \| 8 \| 0.88 \| 0.88 \| 0.88 \| 0.87 \| \|  \| Viridiplantae \| cTP \| 227 \| 0.91 \| 0.87 \| 0.89 \| 0.88 \| \|  \| Viridiplantae \| luTP \| 45 \| 0.76 \| 0.76 \| 0.76 \| 0.75 \| \|  \| Viridiplantae \| noTP \| 1802 \| 0.98 \| 0.99 \| 0.98 \| 0.94 \| \|  \| Metazoa \| noTP \| 5354 \| 0.99 \| 0.99 \| 0.99 \| 0.97 \| \|  \| Fungi \| noTP \| 2263 \| 0.99 \| 0.99 \| 0.99 \| 0.88 \| \|  \| Other \| noTP \| 118 \| 0.98 \| 0.99 \| 0.99 \| 0.95 \| |
| --- | --- | --- | --- | --- | --- | --- | --- | --- | --- | --- | --- | --- | --- | --- | --- | --- | --- | --- | --- | --- | --- | --- | --- | --- | --- | --- | --- | --- | --- | --- | --- | --- | --- | --- | --- | --- | --- | --- | --- | --- | --- | --- | --- | --- | --- | --- | --- | --- | --- | --- | --- | --- | --- | --- | --- | --- | --- | --- | --- | --- | --- | --- | --- | --- | --- | --- | --- | --- | --- | --- | --- | --- | --- | --- | --- | --- | --- | --- | --- | --- | --- | --- | --- | --- | --- | --- | --- | --- | --- | --- | --- | --- | --- | --- | --- | --- | --- | --- | --- | --- | --- | --- | --- | --- | --- | --- | --- | --- | --- | --- | --- | --- | --- | --- | --- | --- | --- | --- | --- | --- |
