# Supplementary material for: Detecting sequence signals in targeting peptides using deep learning
Source: Life Sci Alliance. 2019 Sep 30;2(5):e201900429. doi: 10.26508/lsa.201900429 (PMC6769257; doi:10.26508/lsa.201900429)
Supplement: Supplementary file 5 [file LSA-2019-00429_TableS5.docx]

Table S5: Performance for the cleavage site predictions. This table shows the Recall (accuracy) in the test set yield by each predictor for Mitochondria (mTP), Chloroplast (cTP) , Thylakoid (luTP) and Signal Peptide(SP). The column Recall -5/+5 shows the performance when a positional shift of up to five residues is allowed.

| \|  \| Tool \| Loc \| Recall \| No. Correct \| No. Correct -5/+5 \| Recall -5/+5 \| Total No. \|  \|  \| \| --- \| --- \| --- \| --- \| --- \| --- \| --- \| --- \| --- \| --- \| \|  \| TargetP 2.0 \| SP \| 0.83 \| 2248 \| 2584 \| 0.96 \| 2697 \|  \|  \| \|  \| TargetP 1.1 \| SP \| 0.83 \| 2250 \| 2551 \| 0.95 \| 2697 \|  \|  \| \|  \| PredSL \| SP \| 0.70 \| 1889 \| 2312 \| 0.86 \| 2697 \|  \|  \| \|  \| SignalP 5.0 \| SP \| 0.86 \| 2313 \| 2592 \| 0.96 \| 2697 \|  \|  \| \|  \| TargetP 2.0 \| mTP \| 0.46 \| 230 \| 326 \| 0.65 \| 499 \|  \|  \| \|  \| TargetP 1.1 \| mTP \| 0.42 \| 211 \| 276 \| 0.55 \| 499 \|  \|  \| \|  \| PredSL \| mTP \| 0.16 \| 81 \| 196 \| 0.39 \| 499 \|  \|  \| \|  \| TPpred 3 \| mTP \| 0.39 \| 195 \| 259 \| 0.52 \| 499 \|  \|  \| \|  \| Mitofates \| mTP \| 0.18 \| 88 \| 251 \| 0.50 \| 499 \|  \|  \| \|  \| TargetP 2.0 \| cTP \| 0.49 \| 111 \| 164 \| 0.72 \| 227 \|  \|  \| \|  \| TargetP 1.1 \| cTP \| 0.07 \| 17 \| 104 \| 0.46 \| 227 \|  \|  \| \|  \| PredSL \| cTP \| 0.11 \| 25 \| 67 \| 0.30 \| 227 \|  \|  \| \|  \| TPpred 3 \| cTP \| 0.30 \| 67 \| 105 \| 0.46 \| 227 \|  \|  \| \|  \| TargetP 2.0 \| luTP \| 0.60 \| 27 \| 31 \| 0.69 \| 45 \|  \|  \| \|  \| PredSL \| luTP \| 0.10 \| 5 \| 32 \| 0.71 \| 45 \|  \|  \| |
| --- | --- | --- | --- | --- | --- | --- | --- | --- | --- | --- | --- | --- | --- | --- | --- | --- | --- | --- | --- | --- | --- | --- | --- | --- | --- | --- | --- | --- | --- | --- | --- | --- | --- | --- | --- | --- | --- | --- | --- | --- | --- | --- | --- | --- | --- | --- | --- | --- | --- | --- | --- | --- | --- | --- | --- | --- | --- | --- | --- | --- | --- | --- | --- | --- | --- | --- | --- | --- | --- | --- | --- | --- | --- | --- | --- | --- | --- | --- | --- | --- | --- | --- | --- | --- | --- | --- | --- | --- | --- | --- | --- | --- | --- | --- | --- | --- | --- | --- | --- | --- | --- | --- | --- | --- | --- | --- | --- | --- | --- | --- | --- | --- | --- | --- | --- | --- | --- | --- | --- | --- | --- | --- | --- | --- | --- | --- | --- | --- | --- | --- | --- | --- | --- | --- | --- | --- | --- | --- | --- | --- | --- | --- | --- | --- | --- | --- | --- | --- | --- | --- | --- | --- | --- | --- | --- | --- | --- | --- | --- | --- |
