# Supplementary material for: Detecting sequence signals in targeting peptides using deep learning
Source: Life Sci Alliance. 2019 Sep 30;2(5):e201900429. doi: 10.26508/lsa.201900429 (PMC6769257; doi:10.26508/lsa.201900429)
Supplement: Supplementary file 6 [file LSA-2019-00429_TableS6.docx]

Table S6: Corrected number of proteins annotated with different targeting peptides for the A. thaliana genome using the confusion matrix from Table [S3](supplementary/index.html#table:cm_plant).

| \|  \| Class \| Original \| Corrected \| Shift \| \| --- \| --- \| --- \| --- \| --- \| \|  \| SP \| 4115 \| 4109.3 \| -0.1% \| \|  \| mTP \| 1095 \| 1106.5 \| +1.1% \| \|  \| cTP \| 1448 \| 1410.6 \| -2.6% \| \|  \| luTP \| 127 \| 158.6 \| +24.9% \| |
| --- | --- | --- | --- | --- | --- | --- | --- | --- | --- | --- | --- | --- | --- | --- | --- | --- | --- | --- | --- | --- | --- | --- | --- | --- | --- |
