# Supplementary material for: Detecting sequence signals in targeting peptides using deep learning
Source: Life Sci Alliance. 2019 Sep 30;2(5):e201900429. doi: 10.26508/lsa.201900429 (PMC6769257; doi:10.26508/lsa.201900429)
Supplement: Supplementary file 7 [file LSA-2019-00429_TableS7.docx]

Table S7: The table shows the the agreement with Uniprot annotations. The column fractions contain the number of TargetP 2.0 predictions that agree with the UniProt annotations.

| \| Kingdom \| Organism \| Reference \| TargetP 2.0 \| Uniprot \| Agree \| Fractions \| \| --- \| --- \| --- \| --- \| --- \| --- \| --- \| \| SP \| \| \| \| \| \| \| \| Metazoa \| H. sapiens \| 20585 \| 3698 \| 3521 \| 3382 \| 91.5% \| \| Metazoa \| D. melanogaster \| 13785 \| 3323 \| 3076 \| 2994 \| 90.1% \| \| Metazoa \| M. musculus \| 22286 \| 4278 \| 4042 \| 3883 \| 90.8% \| \| Metazoa \| C. elegans \| 19986 \| 4591 \| 4078 \| 3967 \| 86.4% \| \| Metazoa \| X. tropicalis \| 24138 \| 2366 \| 1933 \| 1841 \| 77.8% \| \| Metazoa \| D. rerio \| 25747 \| 4399 \| 3808 \| 3651 \| 82.9% \| \| Fungi \| S. cerevisiae \| 6049 \| 386 \| 298 \| 272 \| 70.5% \| \| Fungi \| S. pombe \| 5142 \| 252 \| 214 \| 195 \| 77.4% \| \| Viridiplantae \| A. thaliana \| 27623 \| 4115 \| 3543 \| 3374 \| 82.0% \| \| Viridiplantae \| B. distachyon \| 34230 \| 3987 \| 3567 \| 3216 \| 80.7% \| \| Viridiplantae \| O. sativa \| 43588 \| 4687 \| 4169 \| 3644 \| 77.7% \| \| Viridiplantae \| S. lycopersicum \| 33952 \| 3904 \| 2848 \| 2675 \| 68.5% \| \| Viridiplantae \| V. vinifera \| 29882 \| 2980 \| 2199 \| 2019 \| 67.8% \| \| mTP \| \| \| \| \| \| \| \| Metazoa \| H. sapiens \| 20585 \| 627 \| 540 \| 442 \| 70.5% \| \| Metazoa \| D. melanogaster \| 13785 \| 522 \| 136 \| 102 \| 19.5% \| \| Metazoa \| M. musculus \| 22286 \| 631 \| 519 \| 429 \| 68.0% \| \| Metazoa \| C. elegans \| 19986 \| 447 \| 116 \| 88 \| 19.7% \| \| Metazoa \| X. tropicalis \| 24138 \| 453 \| 51 \| 37 \| 8.2% \| \| Metazoa \| D. rerio \| 25747 \| 626 \| 98 \| 70 \| 11.2% \| \| Fungi \| S. cerevisiae \| 6049 \| 368 \| 365 \| 284 \| 77.2% \| \| Fungi \| S. pombe \| 5142 \| 250 \| 266 \| 159 \| 63.6% \| \| Viridiplantae \| A. thaliana \| 27623 \| 1095 \| 526 \| 432 \| 39.5% \| \| Viridiplantae \| B. distachyon \| 34230 \| 970 \| 0 \| 0 \| 0.0% \| \| Viridiplantae \| O. sativa \| 43588 \| 1100 \| 86 \| 67 \| 6.1% \| \| Viridiplantae \| S. lycopersicum \| 33952 \| 931 \| 4 \| 4 \| 0.4% \| \| Viridiplantae \| V. vinifera \| 29882 \| 725 \| 0 \| 0 \| 0.0% \| \| cTP \| \| \| \| \| \| \| \| Viridiplantae \| A. thaliana \| 27623 \| 1448 \| 1222 \| 884 \| 61.0% \| \| Viridiplantae \| B. distachyon \| 34230 \| 1781 \| 0 \| 0 \| 0.0% \| \| Viridiplantae \| O. sativa \| 43588 \| 2049 \| 340 \| 279 \| 13.6% \| \| Viridiplantae \| S. lycopersicum \| 33952 \| 1274 \| 78 \| 57 \| 4.5% \| \| Viridiplantae \| V. vinifera \| 29882 \| 1125 \| 3 \| 2 \| 0.2% \| \| luTP \| \| \| \| \| \| \| \| Viridiplantae \| A. thaliana \| 27623 \| 127 \| 72 \| 58 \| 45.7% \| \| Viridiplantae \| B. distachyon \| 34230 \| 85 \| 0 \| 0 \| 0.0% \| \| Viridiplantae \| O. sativa \| 43588 \| 84 \| 9 \| 5 \| 6.0% \| \| Viridiplantae \| S. lycopersicum \| 33952 \| 117 \| 1 \| 1 \| 0.9% \| \| Viridiplantae \| V. vinifera \| 29882 \| 91 \| 0 \| 0 \| 0.0% \| |
| --- | --- | --- | --- | --- | --- | --- | --- | --- | --- | --- | --- | --- | --- | --- | --- | --- | --- | --- | --- | --- | --- | --- | --- | --- | --- | --- | --- | --- | --- | --- | --- | --- | --- | --- | --- | --- | --- | --- | --- | --- | --- | --- | --- | --- | --- | --- | --- | --- | --- | --- | --- | --- | --- | --- | --- | --- | --- | --- | --- | --- | --- | --- | --- | --- | --- | --- | --- | --- | --- | --- | --- | --- | --- | --- | --- | --- | --- | --- | --- | --- | --- | --- | --- | --- | --- | --- | --- | --- | --- | --- | --- | --- | --- | --- | --- | --- | --- | --- | --- | --- | --- | --- | --- | --- | --- | --- | --- | --- | --- | --- | --- | --- | --- | --- | --- | --- | --- | --- | --- | --- | --- | --- | --- | --- | --- | --- | --- | --- | --- | --- | --- | --- | --- | --- | --- | --- | --- | --- | --- | --- | --- | --- | --- | --- | --- | --- | --- | --- | --- | --- | --- | --- | --- | --- | --- | --- | --- | --- | --- | --- | --- | --- | --- | --- | --- | --- | --- | --- | --- | --- | --- | --- | --- | --- | --- | --- | --- | --- | --- | --- | --- | --- | --- | --- | --- | --- | --- | --- | --- | --- | --- | --- | --- | --- | --- | --- | --- | --- | --- | --- | --- | --- | --- | --- | --- | --- | --- | --- | --- | --- | --- | --- | --- | --- | --- | --- | --- | --- | --- | --- | --- | --- | --- | --- | --- | --- | --- | --- | --- | --- | --- | --- | --- | --- | --- | --- | --- | --- | --- | --- | --- | --- | --- | --- | --- | --- | --- | --- | --- | --- | --- | --- | --- | --- | --- | --- | --- | --- | --- | --- | --- | --- | --- | --- | --- | --- | --- | --- | --- | --- | --- | --- | --- | --- | --- | --- | --- | --- | --- | --- | --- | --- | --- | --- | --- | --- | --- |
