# Supplementary material for: Detecting sequence signals in targeting peptides using deep learning
Source: Life Sci Alliance. 2019 Sep 30;2(5):e201900429. doi: 10.26508/lsa.201900429 (PMC6769257; doi:10.26508/lsa.201900429)
Supplement: Supplementary file 8 [file LSA-2019-00429_TableS8.docx]

Table S8: Frequencies in position two in the test set divided by Viridiplantae (P), Metazoa (M), Fungi (F) and other Eukaryotic organisms (O) sequences for the five categories of proteins, mitochondrial Transit Peptides (mTPs), Signal Peptide (SP), choroplast Transit Peptides (cTP), thylakoid lumenal Tranist Peptides (luTP) and proteins with no targeting peptide (noTP). All frequencies higher than 10% are marked in bold. The top part shows the frequency of the short-chained amino acids that can be cleaved by MAPs. The SUM line is the sum of all these short-chained amino acids, and the Total line is the number of proteins in each class.

| \|  \| AA \| noTP-P \| noTP-M \| noTP-F \| noTP-O \| SP-P \| SP-M \| SP-F \| SP-O \| mTP-P \| mTP-M \| mTP-F \| mTP-O \| cTP \| luTP \| \| --- \| --- \| --- \| --- \| --- \| --- \| --- \| --- \| --- \| --- \| --- \| --- \| --- \| --- \| --- \| --- \| \|  \| Ala \| 21.7% \| 23.7% \| 12.4% \| 16.0% \| 47.7% \| 15.7% \| 6.9% \| 10.0% \| 40.8% \| 40.2% \| 6.3% \| 0.0% \| 71.3% \| 71.1% \| \|  \| Cys \| 0.4% \| 0.6% \| 0.4% \| 0.0% \| 0.0% \| 0.8% \| 0.0% \| 0.0% \| 0.0% \| 0.0% \| 0.0% \| 0.0% \| 0.0% \| 0.0% \| \|  \| Gly \| 10.6% \| 6.1% \| 5.5% \| 4.2% \| 6.9% \| 8.1% \| 4.1% \| 0.0% \| 1.5% \| 0.8% \| 1.1% \| 0.0% \| 1.4% \| 0.0% \| \|  \| Pro \| 3.1% \| 6.0% \| 5.4% \| 3.4% \| 0.4% \| 2.2% \| 2.1% \| 0.0% \| 0.8% \| 0.8% \| 1.1% \| 0.0% \| 0.0% \| 0.0% \| \|  \| Ser \| 12.8% \| 14.4% \| 26.6% \| 16.8% \| 4.0% \| 4.1% \| 4.1% \| 6.7% \| 9.2% \| 4.3% \| 12.6% \| 12.5% \| 10.6% \| 8.9% \| \|  \| Thr \| 3.3% \| 4.5% \| 7.6% \| 5.9% \| 2.9% \| 3.2% \| 0.7% \| 0.0% \| 1.5% \| 1.2% \| 2.1% \| 0.0% \| 2.3% \| 2.2% \| \|  \| Val \| 5.1% \| 4.4% \| 4.9% \| 3.4% \| 4.3% \| 3.4% \| 9.0% \| 0.0% \| 1.5% \| 2.4% \| 0.0% \| 0.0% \| 1.4% \| 4.4% \| \|  \| SUM \| 57% \| 59.7% \| 62.8% \| 49.7% \| 66.2% \| 37.5% \| 26.9% \| 16.7% \| 55.3% \| 49.7% \| 23.2% \| 12.5% \| 87% \| 86.6% \| \|  \| Asp \| 8.5% \| 7.8% \| 7.1% \| 6.7% \| 1.1% \| 2.7% \| 0.7% \| 0.0% \| 0.0% \| 0.0% \| 0.0% \| 0.0% \| 0.5% \| 0.0% \| \|  \| Glu \| 13.1% \| 10.5% \| 5.9% \| 11.8% \| 5.8% \| 4.8% \| 0.7% \| 0.0% \| 0.0% \| 0.0% \| 0.0% \| 0.0% \| 3.7% \| 2.2% \| \|  \| Phe \| 1.2% \| 1.7% \| 1.8% \| 3.4% \| 0.0% \| 1.3% \| 4.8% \| 0.0% \| 5.4% \| 6.7% \| 12.6% \| 12.5% \| 0.0% \| 0.0% \| \|  \| His \| 0.4% \| 0.9% \| 0.7% \| 0.0% \| 1.1% \| 1.4% \| 1.4% \| 0.0% \| 1.5% \| 0.8% \| 1.1% \| 0.0% \| 0.0% \| 2.2% \| \|  \| Ile \| 2.0% \| 1.4% \| 1.8% \| 4.2% \| 2.5% \| 2.2% \| 4.1% \| 3.3% \| 3.1% \| 2.4% \| 4.2% \| 12.5% \| 0.9% \| 0.0% \| \|  \| Lys \| 5.2% \| 3.4% \| 4.7% \| 5.9% \| 10.8% \| 19.5% \| 17.9% \| 46.7% \| 7.7% \| 1.2% \| 5.3% \| 0.0% \| 0.5% \| 0.0% \| \|  \| Leu \| 1.8% \| 3.3% \| 3.3% \| 4.2% \| 1.1% \| 5.4% \| 9.7% \| 3.3% \| 5.4% \| 28.7% \| 38.9% \| 25.0% \| 2.8% \| 0.0% \| \|  \| Met \| 2.5% \| 1.6% \| 1.1% \| 1.7% \| 2.9% \| 2.9% \| 4.1% \| 0.0% \| 4.6% \| 0.4% \| 2.1% \| 0.0% \| 2.3% \| 2.2% \| \|  \| Asn \| 2.5% \| 3.7% \| 5.4% \| 6.7% \| 3.6% \| 5.0% \| 4.1% \| 20.0% \| 1.5% \| 0.0% \| 1.1% \| 0.0% \| 0.5% \| 0.0% \| \|  \| Gln \| 1.6% \| 2.0% \| 1.9% \| 0.0% \| 0.0% \| 2.5% \| 8.3% \| 0.0% \| 3.8% \| 2.8% \| 3.2% \| 12.5% \| 1.4% \| 4.4% \| \|  \| Arg \| 2.6% \| 2.7% \| 2.4% \| 5.0% \| 4.7% \| 12.3% \| 15.2% \| 10.0% \| 6.2% \| 2.4% \| 3.2% \| 25.0% \| 0.0% \| 0.0% \| \|  \| Trp \| 0.1% \| 0.6% \| 0.2% \| 0.0% \| 0.4% \| 1.7% \| 0.0% \| 0.0% \| 2.3% \| 3.5% \| 2.1% \| 0.0% \| 0.0% \| 2.2% \| \|  \| Tyr \| 1.4% \| 0.7% \| 0.8% \| 0.8% \| 0.0% \| 0.9% \| 2.1% \| 0.0% \| 3.1% \| 1.6% \| 3.2% \| 0.0% \| 0.5% \| 0.0% \| \|  \| Total \| 1802 \| 5354 \| 2263 \| 118 \| 282 \| 2251 \| 133 \| 31 \| 125 \| 263 \| 103 \| 8 \| 227 \| 45 \| |
| --- | --- | --- | --- | --- | --- | --- | --- | --- | --- | --- | --- | --- | --- | --- | --- | --- | --- | --- | --- | --- | --- | --- | --- | --- | --- | --- | --- | --- | --- | --- | --- | --- | --- | --- | --- | --- | --- | --- | --- | --- | --- | --- | --- | --- | --- | --- | --- | --- | --- | --- | --- | --- | --- | --- | --- | --- | --- | --- | --- | --- | --- | --- | --- | --- | --- | --- | --- | --- | --- | --- | --- | --- | --- | --- | --- | --- | --- | --- | --- | --- | --- | --- | --- | --- | --- | --- | --- | --- | --- | --- | --- | --- | --- | --- | --- | --- | --- | --- | --- | --- | --- | --- | --- | --- | --- | --- | --- | --- | --- | --- | --- | --- | --- | --- | --- | --- | --- | --- | --- | --- | --- | --- | --- | --- | --- | --- | --- | --- | --- | --- | --- | --- | --- | --- | --- | --- | --- | --- | --- | --- | --- | --- | --- | --- | --- | --- | --- | --- | --- | --- | --- | --- | --- | --- | --- | --- | --- | --- | --- | --- | --- | --- | --- | --- | --- | --- | --- | --- | --- | --- | --- | --- | --- | --- | --- | --- | --- | --- | --- | --- | --- | --- | --- | --- | --- | --- | --- | --- | --- | --- | --- | --- | --- | --- | --- | --- | --- | --- | --- | --- | --- | --- | --- | --- | --- | --- | --- | --- | --- | --- | --- | --- | --- | --- | --- | --- | --- | --- | --- | --- | --- | --- | --- | --- | --- | --- | --- | --- | --- | --- | --- | --- | --- | --- | --- | --- | --- | --- | --- | --- | --- | --- | --- | --- | --- | --- | --- | --- | --- | --- | --- | --- | --- | --- | --- | --- | --- | --- | --- | --- | --- | --- | --- | --- | --- | --- | --- | --- | --- | --- | --- | --- | --- | --- | --- | --- | --- | --- | --- | --- | --- | --- | --- | --- | --- | --- | --- | --- | --- | --- | --- | --- | --- | --- | --- | --- | --- | --- | --- | --- | --- | --- | --- | --- | --- | --- | --- | --- | --- | --- | --- | --- | --- | --- | --- | --- | --- | --- | --- | --- | --- | --- | --- | --- | --- | --- | --- | --- | --- | --- | --- | --- | --- | --- | --- | --- | --- | --- | --- | --- | --- | --- | --- | --- | --- | --- | --- | --- | --- | --- | --- | --- | --- | --- | --- | --- | --- | --- | --- | --- | --- | --- | --- | --- | --- | --- | --- | --- |
