# Supplementary material for: Detecting sequence signals in targeting peptides using deep learning
Source: Life Sci Alliance. 2019 Sep 30;2(5):e201900429. doi: 10.26508/lsa.201900429 (PMC6769257; doi:10.26508/lsa.201900429)
Supplement: Supplementary file 9 [file LSA-2019-00429_TableS9.docx]

Table S9: Amino acid frequencies in position two in A. thaliana for proteins predicted to belong to different classes by TargetP 2.0. All frequencies higher than 10% are marked in bold.

| \|  \| AA \| noTP \| SP \| mTP \| cTP \| luTP \| \| --- \| --- \| --- \| --- \| --- \| --- \| --- \| \|  \| A \| **16.4%** \| **25.7%** \| **27.4%** \| **48.3%** \| **32.1%** \| \|  \| C \| 1.0% \| 0.7% \| 0.4% \| 0.7% \| 3.6% \| \|  \| D \| 8.1% \| 3.8% \| 0.0% \| 1.0% \| 3.6% \| \|  \| E \| **13.0%** \| 8.5% \| 0.8% \| 5.0% \| **10.7%** \| \|  \| F \| 2.1% \| 2.6% \| 6.6% \| 0.7% \| 3.6% \| \|  \| G \| **11.0%** \| 7.6% \| 1.9% \| 2.0% \| 0.0 % \| \|  \| H \| 0.9% \| 0.7% \| 0.8% \| % \| 3.6% \| \|  \| I \| 2.6% \| 2.7% \| 3.1% \| 2.7% \| 3.6% \| \|  \| K \| 5.2% \| **11.1%** \| 3.5% \| 1.3% \| 3.6% \| \|  \| L \| 3.5% \| 2.9% \| **11.6%** \| 6.0% \| 3.6% \| \|  \| M \| 2.4% \| 2.5% \| 3.5% \| 2.0% \| 3.6% \| \|  \| N \| 3.5% \| 3.4% \| 3.5% \| 1.3% \| 3.6% \| \|  \| P \| 2.6% \| 1.0% \| 0.4% \| 1.3% \| 3.6% \| \|  \| Q \| 1.8% \| 1.1% \| 3.1% \| 1.7% \| 3.6% \| \|  \| R \| 2.9% \| 2.9% \| 7.7% \| 0.3% \| 0.0 % \| \|  \| S \| **10.1%** \| 9.3% \| **15.4%** \| **14.8%** \| 3.6% \| \|  \| T \| 4.4% \| 5.8% \| 2.7% \| 4.0% \| 3.6% \| \|  \| V \| 6.2% \| 6.2% \| 2.3% \| 4.4% \| 7.1% \| \|  \| W \| 0.8% \| 0.5% \| 1.9% \| 1.0% \| 0.0 % \| \|  \| Y \| 1.4% \| 0.8% \| 3.5% \| 1.3% \| 3.6% \| |
| --- | --- | --- | --- | --- | --- | --- | --- | --- | --- | --- | --- | --- | --- | --- | --- | --- | --- | --- | --- | --- | --- | --- | --- | --- | --- | --- | --- | --- | --- | --- | --- | --- | --- | --- | --- | --- | --- | --- | --- | --- | --- | --- | --- | --- | --- | --- | --- | --- | --- | --- | --- | --- | --- | --- | --- | --- | --- | --- | --- | --- | --- | --- | --- | --- | --- | --- | --- | --- | --- | --- | --- | --- | --- | --- | --- | --- | --- | --- | --- | --- | --- | --- | --- | --- | --- | --- | --- | --- | --- | --- | --- | --- | --- | --- | --- | --- | --- | --- | --- | --- | --- | --- | --- | --- | --- | --- | --- | --- | --- | --- | --- | --- | --- | --- | --- | --- | --- | --- | --- | --- | --- | --- | --- | --- | --- | --- | --- | --- | --- | --- | --- | --- | --- | --- | --- | --- | --- | --- | --- | --- | --- | --- | --- | --- | --- | --- | --- |
